# Reshaping internationalization of medical education in 2023

**DOI:** 10.1186/s12909-023-04374-2

**Published:** 2023-05-23

**Authors:** Anette Wu

**Affiliations:** Department of Medicine and Pathology and Cell Biology, Vagelos College of Physicians and Surgeons, Columbia University, New York, NY USA

## Abstract

The global COVID-19 pandemic has shown the need for internationalization of medical education, in order to facilitate global collaborative problem solving in healthcare. In 2023, it is time to reshape IoME within the context of our time, and share new visions, ideas, and formats. This collection of articles reports on theories and actions in IoME.

## Main Text

The global COVID-19 pandemic has shown that the improvement of healthcare for all people worldwide is a collaborative effort that requires transnational connection and actions. This provides an opportunity for expanding efforts in internationalization of medical education (IoME).

What should medical educators understand the term IoME to mean?

In line with the broader definition of internationalization of higher education, it can best be described as the *process of purposefully integrating international, intercultural, or global dimensions into medical education in order to enhance its quality and prepare all graduates for professional practice in a globalized world* [1, 2]. As a result, physicians may regard themselves as part of a worldwide medical community and solve healthcare issues globally in a collaborative manner. Thus, IoME promotes international healthcare understanding and cooperation, minimizes healthcare nationalism, and equitably improves the health of all people worldwide [3].

Internationalization in higher education has its roots going back centuries. However, IoME is still a relatively new area of modern medical education. Research and published work are lacking in the global medical literature [1]. IoME is often confused and interchangeably used with the term of, and research in Global Health education. IoME should not be equated with Global Health education as the latter often represents a narrow focus on the low- and middle-income countries (LMIC). Although IoME is a global phenomenon, the understandings and perspectives of the Global North have traditionally dominated, and therefore it addresses only a narrow spectrum of activities transpiring globally.

IoME should not solely focus on programmatic efforts but should increasingly include studies of theories, concepts, and formats, plus define competencies and goals. Scholarly research on longitudinal outcomes of these actions, along with work and research on globalization of medical education to include medical curricula and teaching material, is needed.

Motivations for IoME have focused on three major models. The first two, the market and social transformation models, have their limitations and respectively involve competition for leadership in science and clinical care via global ranking, or focus on health equity – often geared towards the LMIC. The lesser known liberal model includes the ambassadorial role of students to promote international understanding and collaboration and is known as “soft diplomacy” or “science diplomacy” [4].

Formats to date are dominated by reports on international partnerships, student mobility, and internationalization “at home” (IaH) and/or internationalization of the curriculum.

IoME (and IHE) is heavily intertwined with political and societal influences and undergoes constant change. In 2023, like many other areas of medical education, IoME has been significantly impacted by the aftermath of the pandemic:

1. National political, societal, and socio-economic, challenges resulted in an inward focus in many countries leading to deglobalization and nationalization trends in healthcare. This was accompanied by an (expectedly transient) inward shift of policies and societal resources—at a time when IoME is needed more than ever [5, 6].
2. The rise in global conflicts and wars, and the overall rise in populism in many countries limits opportunities and resources due to lack of motivation to support internationalization efforts and IoME. The above calls for reconsidering the need of the “liberal model” as motivation for IoME.
3. New challenges in medical education affect medicine in the post COVID-19 pandemic era and will ultimately impact IoME and global collaboration in medicine [7]. Over the last decade, even prior to COVID-19, medical education experienced significant change(s). A recent report in *Lancet* by Frenk and colleagues [7] outlined important elements for upcoming changes and suggested new pathways in medical education that will need to be integrated in the learners’ portfolio [7]. International collaboration in medical education and IoME will need to be integrated into these changes.
4. Further globalization in many countries may potentially reshuffle leadership in science and medicine in the future.
5. New technology, digitalization, and the impending arrival of artificial intelligence (“Chat GPT”) will determine how medicine is practiced and how global collaborations and international education will be included.
6. Altered mobility caused by forced migration due to climate change and conflicts will shift patient populations, alter the global healthcare workforce, and change the landscape of medical education as a whole.
7. Raising awareness and concerns about the environment and climate change will soon shape how IoME and student mobility will be practiced and executed.
8. Furthermore, issues regarding societal diversity, equity, and inclusion, and discussion about decolonization in medicine globally and the ethics of student mobility in IoME will dominate new ideas in IoME and reshape old endeavors.

All of the above will have an impact on how IoME will be practiced, studied, and shaped in 2023. It is time to revisit IoME as a complex product, within the context of our time, and share new visions, ideas, and formats.

This current collection of articles invites scholars, medical educators, students, administrators and leadership to report on theories and actions in IoME, and to share regional, national, and global experiences in IoME from an interdisciplinary and diverse perspective. We invite the private sector to share their views and visions. Topics can be non-traditional (e.g., challenging English as the leading scientific language, issues regarding IoME in times of war and political conflicts, etc.). We also hope that our collection of articles serves as a baseline for longitudinal outcomes research on healthcare.

At a time of major change(s) in medical education this collection is meant to provide a framework for establishing and reshaping the field of IoME as a research area for scholarly work and sharing of programmatic efforts – to exchange knowledge internationally, which is the cornerstone of any work in IHE. IoME must become a part of medical education, in order to ensure that worldwide healthcare issues can be solved collaboratively in the future. In our interconnected world, IoME must find its place in medical education for the betterment of the health of all people worldwide.

## Data Availability

Not applicable.

## Abbreviations

IoME: Internationalization of Medical Education
IHE: International Higher Education
LMIC: Low- and middle-Income countries
